# Postnatal Dendritic Growth and Spinogenesis of Layer-V Pyramidal Cells Differ between Visual, Inferotemporal, and Prefrontal Cortex of the Macaque Monkey

**DOI:** 10.3389/fnins.2017.00118

**Published:** 2017-03-13

**Authors:** Tomofumi Oga, Guy N. Elston, Ichiro Fujita

**Affiliations:** ^1^Laboratory for Cognitive Neuroscience, Graduate School of Frontier Biosciences, Osaka UniversitySuita, Japan; ^2^Centre for Cognitive NeuroscienceSunshine Coast, QLD, Australia; ^3^Center for Information and Neural Networks, National Institute of Information and Communications Technology and Osaka UniversitySuita, Japan

**Keywords:** postnatal development, visual cortex, inferior temporal cortex, area 12, area TE, basal dendrite, spine, synaptogenesis

## Abstract

Pyramidal cells in the primate cerebral cortex, particularly those in layer III, exhibit regional variation in both the time course and magnitude of postnatal growth and pruning of dendrites and spines. Less is known about the development of pyramidal cell dendrites and spines in other cortical layers. Here we studied dendritic morphology of layer-V pyramidal cells in primary visual cortex (V1, sensory), cytoarchitectonic area TE in the inferotemporal cortex (sensory association), and granular prefrontal cortex (Walker's area 12, executive) of macaque monkeys at the ages of 2 days, 3 weeks, 3.5 months, and 4.5 years. We found that changes in the basal dendritic field area of pyramidal cells were different across the three areas. In V1, field size became smaller over time (largest at 2 days, half that size at 4.5 years), in TE it did not change, and in area 12 it became larger over time (smallest at 2 days, 1.5 times greater at 4.5 years). In V1 and TE, the total number of branch points in the basal dendritic trees was similar between 2 days and 4.5 years, while in area 12 the number was greater in the adult monkeys than in the younger ones. Spine density peaked at 3 weeks and declined in all areas by adulthood, with V1 exhibiting a faster decline than area TE or area 12. Estimates of the total number of spines in the dendritic trees revealed that following the onset of visual experience, pyramidal cells in V1 lose more spines than they grow, whereas those in TE and area 12 grow more spines than they lose during the same period. These data provide further evidence that the process of synaptic refinement in cortical pyramidal cells differs not only according to time, but also location within the cortex. Furthermore, given the previous finding that layer-III pyramidal cells in all these areas exhibit the highest density and total number of spines at 3.5 months, the current results indicate that pyramidal cells in layers III and V develop spines at different rates.

## Introduction

Pyramidal cells in the primate cerebral cortex are characterized by different rates of growth and atrophy of both their dendrites and spines during development, resulting in marked phenotypic variation in their cellular structure among different areas in the mature brain (Elston et al., 1996, 2009, 2010a,b, 2011b; Elston and Rosa, 1997, 1998; Jacobs et al., 1997, 2001; Petanjek et al., 2008, 2011; Amatrudo et al., 2012; Bianchi et al., 2013). In some cortical areas, such as macaque primary visual cortex (V1), the basal dendritic trees of layer-III pyramidal cells grow to their full extent around birth, then become successively smaller through infancy, adolescence, and adulthood (Boothe et al., 1979; Elston et al., 2009, 2010a). In other cortical areas, such as inferotemporal cortex (posterior dorsal part of cytoarchitectonic area TE) and granular prefrontal cortex (gPFC; area 12; Walker, 1940) in the macaque (Elston et al., 2009, 2010a) and gPFC (Brodmann's area [BA] 9, Brodmann, 1909) in humans (Petanjek et al., 2008, 2011), pyramidal cells grow increasingly larger basal dendritic trees from birth through adulthood; only in older age (>50yrs), dendrites decrease their arbor size (BA10 and BA 12 in human; Jacobs et al., 1997). The numbers of spines grown and pruned from the basal dendritic trees of pyramidal cells also differ considerably among cortical areas. For example, basal dendrites of layer-III pyramidal cells in macaque V1 attain an average of 1900 spines by the age of 3.5 months (Elston et al., 2009, 2010a)—the period corresponding to peak synaptogenesis in the neuropil (Rakic et al., 1986; Bourgeois et al., 1989; Bourgeois and Rakic, 1993). A large proportion of these spines (>75%) are then pruned, resulting in a net reduction of pyramidal cell spines between birth and adulthood (Elston et al., 2009). In contrast, layer-III cells in area TE can grow an average of 10,400 spines by 3.5 months, and those in area 12 as many as 15,900, those in area 12 as many as 15,900, and in both cases there is a net increase in the number of spines between birth and adulthood (Elston et al., 2009, 2010a).

These differences in the developmental profiles of pyramidal cells result in systematic structural differences in the adult macaque and human brain such that pyramidal cells become more spinous as brain regions transition from sensory to association to executive cortex (Elston and Rosa, 1997; Elston et al., 1999a, 2006a; Jacobs et al., 2001; see Elston, 2007; Elston and Fujita, 2014, for review). These anatomical differences have been proposed to provide a basis for specialized physiological and behavioral functions (Jacobs and Scheibel, 2002; Elston, 2007; Spruston, 2008; Amatrudo et al., 2012; Eyal et al., 2016; Mochizuki et al., 2016). However, most of this research was focused on layer III. In the case of layer V, most research has been focussed on a single cortical area (visual cortex: Lund et al., 1977; Boothe et al., 1979; Takashima et al., 1980; Becker et al., 1984; motor cortex: Nakamura et al., 1985; prefrontal cortex: Mrzljak et al., 1992; Koenderink et al., 1994; Anderson et al., 1995; Koenderink and Uylings, 1995; Petanjek et al., 2008, 2011). Whether and how the developmental profiles differ across cortical areas in infragranular layers remains unclear. Furthermore, few studies have compared developmental profiles of pyramidal cells between supragranular and infragranular layers.

Here, we performed a systematic study of the basal dendritic trees of layer-V pyramidal cells in macaque V1, TE, and area 12, thus representing the hierarchical functions of primary sensory, sensory association, and executive cortex, respectively. To characterize growth, spinogenesis, and pruning throughout development and into maturity, each was assessed at four time points, the earliest being the 2nd postnatal day and the latest being at 4.5 years. We conducted experiments in the same animals in which we examined layer-III pyramidal cells in our previous studies (Elston et al., 2009, 2010a,b, 2011b), thus facilitating inter-laminar comparison of the developmental process. We demonstrate that basal dendrites of layer-V cortical pyramidal cells exhibit area- and layer-specific developmental profiles.

## Materials and methods

### Animals and care

Five male macaque monkeys (*Macaca fascicularis*) aged 2 days (D), 3 weeks (W), 3.5 months (M), and 4.5 years (Y) were used in the present study (2 monkeys at 4.5Y) (Table 1). The animal-experiment committee of Osaka University approved the protocols for animal care and experimentation, which were conducted in accordance with the *Guide for the Care and Use of Laboratory Animals* issued by the National Institutes of Health, USA [DHEW Publication No. (NIH) 85–23, Revised 1996, Office of Science and Health Reports, DRR/NIH, Bethesda, MD 20205, USA].

**Table 1:** **The number of cells analyzed for each cortical area/age group**.

Age^*^	**2D**	**3W**	**3.5M**	**4.5Y**	**4.5Y**
Animal	CI9	CI1	CI10	MF1	CI15
Body weight (kg)	0.35	0.56	0.56	–	3.6
V1	23	9	22	16	10
TE	17	37	41	15	11
12	27	35	19	20	9

* *D, W, M, Y: postnatal days, weeks, months, and years. Total number of cells: 311*.

The four time points were chosen because they equate to just after birth, the time including the critical period for ocular dominance shift (Horton and Hocking, 1997), the time of peak synaptogenesis (Rakic et al., 1986; Bourgeois et al., 1989; Bourgeois and Rakic, 1993), and young adulthood, respectively. These ages correspond to those studied for layer-III pyramidal cells in these same cortical areas (Elston et al., 2009, 2010a). Indeed, these data were sampled from the same animals as the layer-III data, allowing us to rule out inter-individual variation as a possible confound in inter-laminar comparisons.

### Intracellular dye injection in lightly fixed tissues

**Figure 1:**
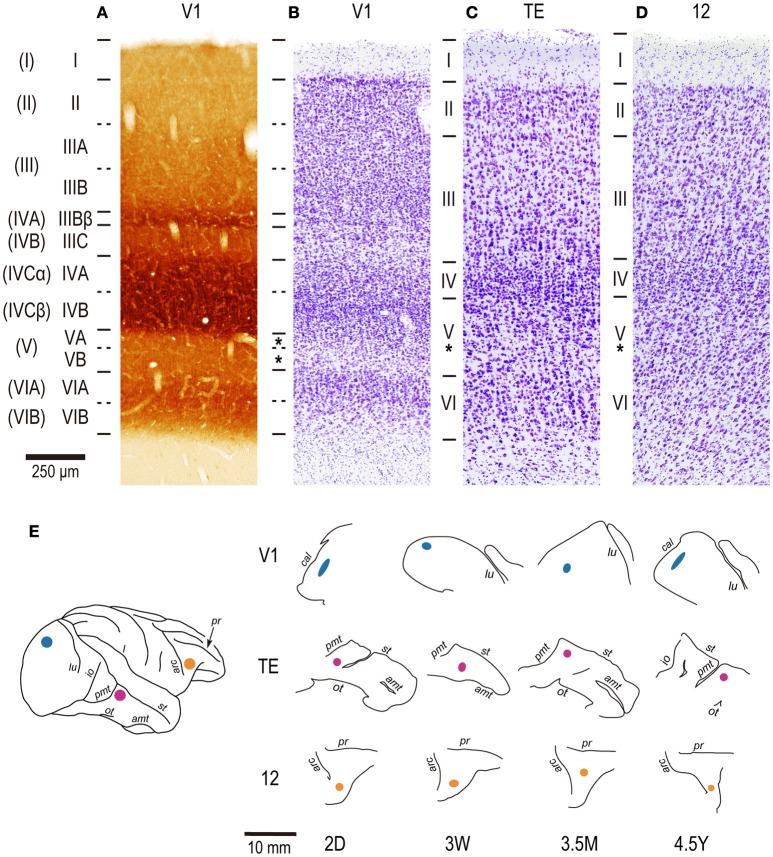
**The layer structure of V1, area TE, and area 12 and the injection sites**. For V1, two consecutive sections were stained by cytochrome oxidase histochemistry **(A)** and cresyl violet for Nissl substance **(B)**. Sections for area TE **(C)** and area 12 **(D)** are stained only for Nissl substance. All sections were from a 2-year old cynomolgus monkey. The nomenclature by Hassler (1966) is listed left, and that by Brodmann (1909) is listed in the far left parentheses. **(E)** Injection sites for individual cases. Injections were made into the operculum for V1, a dorsal part of area TE immediately anterior to the posterior middle temporal sulcus (pmt) for TE, and a ventromedial prefrontal cortex, anterior to the inferior limb of the arcuate sulcus for area 12. All injections were made into layer V (asterisks). Cal, calcarine; lu, lunate; io, inferior occipital; ot, occipito-temporal; amt, anterior middle temporal; st, superior temporal; arc, arcuate; pr, principal. 2D, 3W, 3.5M, 4.5Y: postnatal 2 days, 3 weeks, 3.5 months, and 4.5 years, respectively.

All methods employed in the present study were the same as those detailed in our previous studies on pyramidal cell development (Elston et al., 2009, 2010a,b, 2011b). In brief, following overdose with sodium pentobarbital (>75 mg/kg intravenously or intraperitoneally; Dainippon Sumitomo Pharma, Osaka, Japan) animals were perfused intracardially with 0.9% saline in 0.1 M phosphate buffer (pH = 7.2) and then 4% paraformaldehyde in the same phosphate buffer, followed by removal of the brain. Tissue for V1 was taken from the dorsolateral region of the exposed occipital operculum, corresponding to the central 5–7 degrees of visual representation (Figure 1E; Daniel and Whitteridge, 1961; Oga et al., 2016). We sampled tissue for inferotemporal cortex from the middle third of the inferior temporal gyrus immediately anterior to the posterior middle temporal sulcus (TE; TEp of Seltzer and Pandya, 1978; TEpd of Yukie, 1997). Tissue for prefrontal cortex was taken from the exposed portion of the ventrolateral granular prefrontal cortex (area 12 of Walker, 1940), also known as 12vl (Preuss and Goldman-Rakic, 1991) or 47/12 (Petrides and Pandya, 2002). All tissues were taken from the right hemisphere.

The lightly perfused tissue was flat-mounted as described previously (Elston et al., 2010a) and postfixed overnight between glass slides in 4% paraformaldehyde. Tangential sections (250 μm) were cut the following morning with the aid of a vibratome. To visualize nuclei of individual cells, we incubated the sections in 10^−5^ mol/L of the fluorescent dye 4,6 diamidino-2-phenylindole (DAPI; Sigma D9542, St Louis, USA) in phosphate buffer at room temperature for approximately 10 min before intracellular injection. The DAPI-labeled sections were mounted between two cellulose nitrate membrane filters (AABG02500, Millipore, Bedford, USA), the uppermost having a “window” to allow visualization of the tissue during injection. Injection was performed with a Leica micromanipulator coupled to a fixed-stage microscope (Eclipse FN1; Nikon, Tokyo, Japan) that was equipped with UV excitation (341–343 nm).

**Figure 2:**
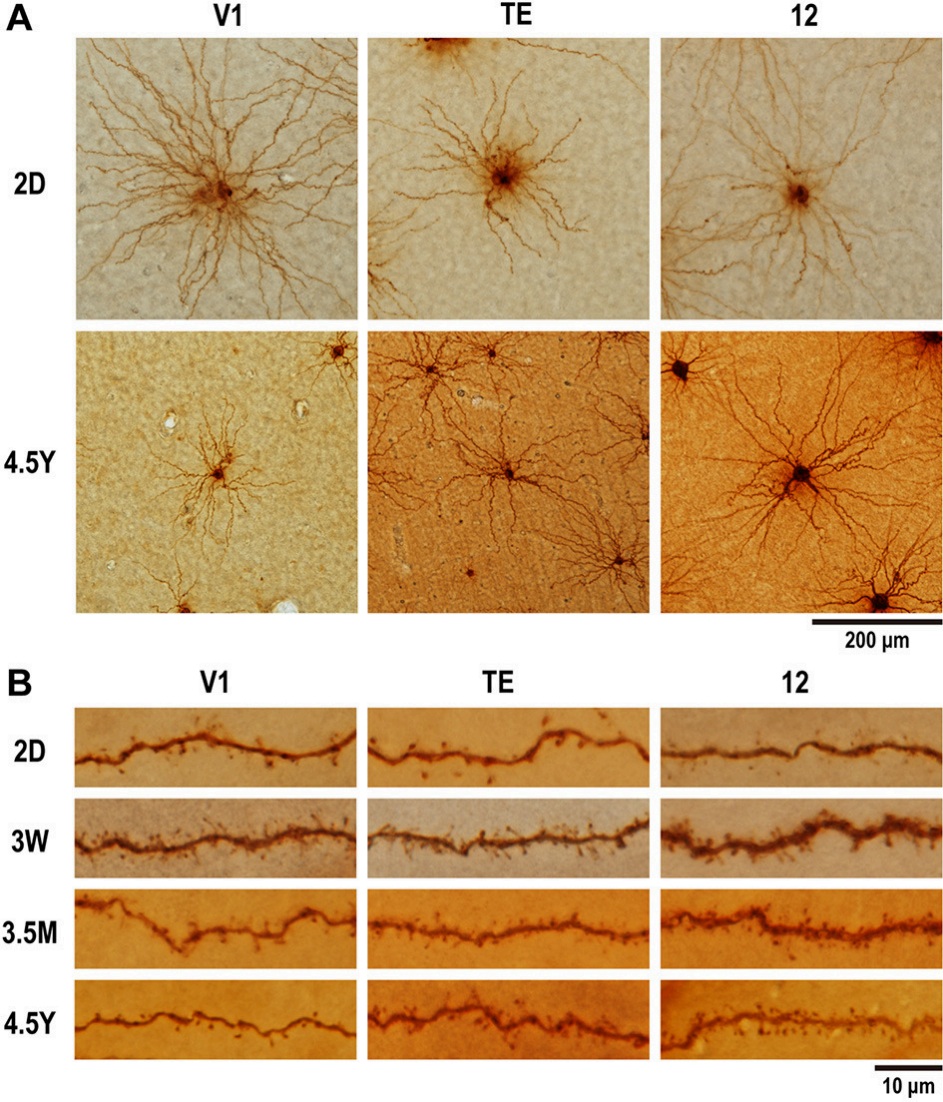
**Photomicrographs of layer-V pyramidal cells individually injected in tangential sections with Lucifer Yellow and reacted for DAB product. (A)** Labeled cells for area V1, area TE, and area 12 at 2 days (2D) and 4.5 years (4.5Y). Dendritic branches of pyramidal cell at 4.5Y was smaller than those at 2D in V1, did not change in area TE, and became greater at 4.5Y than at 2D in area 12. **(B)** Dendritic spines along a dendritic segment in the three areas at each of the four time points. Dendritic segments were poor in spines at 2D, most spinous at 3W in V1, at 3W to 3.5M in area TE and area 12, then spines were pruned toward adulthood.

Although V1, area TE, and area 12 have area-specific laminar structures, all exhibit a distinct granular layer IV (Figures 1A–D). The section which contained layer V was easily identified in DAPI-labeled sections as that immediately below the neuron-dense granular layer. Layer-V pyramidal cells were injected in each slice. Care was taken to select the same region within each cortical area from which we sampled layer-III pyramidal cells in our previous studies (Elston et al., 2009, 2010a), such that cells included in the present study were located immediately below those sampled in layer III. Pyramidal cells were injected under visual guidance with continuous current (up to 100 nA), and the slices were processed for a light-stable reaction product by immunohistochemistry (Figure 2; see Elston and Rosa, 1997; Oga et al., 2016).

### Morphological analysis

We selected neurons for analysis only when their basal dendritic arbors were fully contained within the tissue section (Figure 2A). They were reconstructed using Neurolucida software (MBF Bioscience, Williston, VT, USA) coupled to a microscope (Eclipse 80i; Nikon) that was equipped with a motorized stage (Ludl Electric Products, Hawthorne, NY, USA) and a charge-coupled device camera (CX9000; MBF Biosciences).

**Figure 3:**
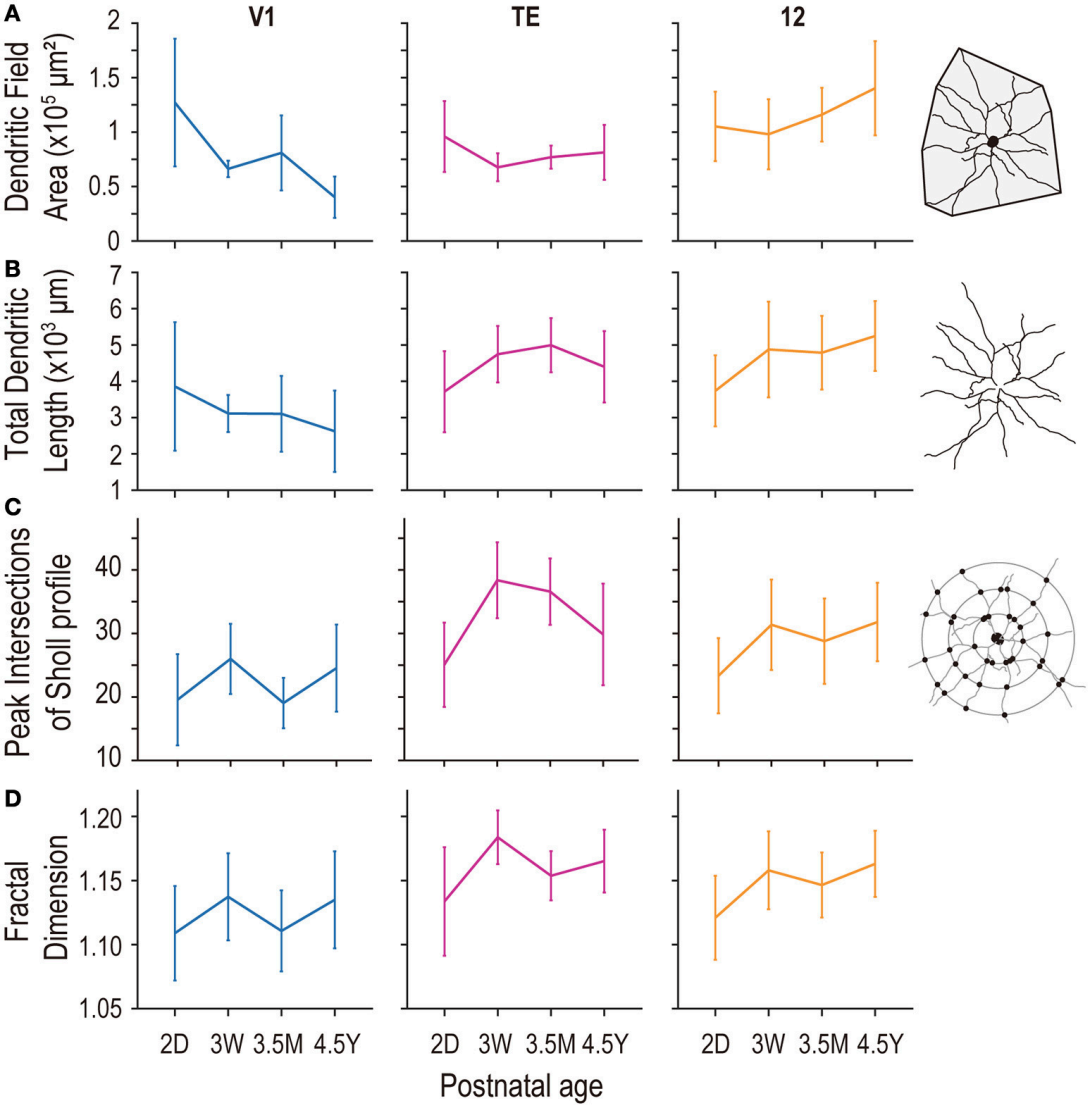
**Plots of the size (A)**, length **(B)**, peak intersections of Sholl analysis **(C)**, and complexity as assessed by fractal dimension **(D)** of the basal dendritic trees of layer V pyramidal cells in area V1, are TE, and area 12 at 2 days (2D), 3.5 months (3.5M), and 4.5 years (4.5Y). Error bars indicate standard deviation.

Dendritic field area was determined in the tangential plane as the area contained within a convex hull traced around the outermost distal dendritic terminations in reconstructions that were collapsed to yield two-dimensional images (see Figure 3A, inset). The cell-body area and total dendritic length were also calculated from these 2-dimensional projections for compatibility with previous studies (see Figure 3B, inset) (e.g., Elston et al., 1996, 2009, 2010a,b, 2011b; Elston and Rosa, 1997, 1998). The branching profiles of dendritic trees were determined by Sholl analysis (Sholl, 1953). In this analysis, we counted intersections between the dendritic arbors and concentric circles. The circles were centered on the cell body with radii incremented at 10-μm steps (see Figure 3C, inset). By plotting the number of intersections against the radii, we obtained the entire Sholl profile for a neuron. The peak value of the profile was used as an index for dendritic branching complexity.

Spines were drawn with a Camera Lucida system at × 100 magnification (numerical aperture: 1.49; CFI Apo TIRF 100 × H/1.49, Nikon), and quantified as a function of distance from the cell body to the distal tips of the dendrites. As with all of our previous studies, we selected horizontally-projecting dendrites for our calculations of spine densities to avoid trigonometric error. Spine densities were calculated per 10-μm interval along the entire length of 10 individually drawn, randomly selected, dendrites in each cortical area for each age group (Eayrs and Goodhead, 1959; Valverde, 1967). We made no distinction between different spine types (e.g., sessile and pedunculated spines; Jones and Powell, 1969). We estimated the total number of spines in the basal dendritic tree of the “average” cell in each area/age combination by combining the Sholl profile and the spine density profile. A value of the Sholl profile at given distance from the cell body indicates the number of 10-μm dendritic segments at the distance. A value of the spine density profile at the distance indicates the number of spines along a single 10-μm dendritic segment. A product of the value of Sholl profile (the number of 10-μm segments at a given Sholl diameter) and the value of spine density profile (the number of spines per 10-μm segment) at the corresponding distance indicates the number of spines at the given distance. Finally, we obtained the total number of spines by summing up the number of spines profile across distance (Elston, 2001).

Given that shrinkage of brain tissue caused by perfusion has been estimated to be small (2.5%; see Oga et al., 2016), and any such shrinkage is consistent with previous cell injection studies, the measured values were not corrected for tissue shrinkage. Statistical analyses were performed with Matlab version 2016a (Mathworks, Inc., Natick, MA, USA).

## Results

We sampled and analyzed 311 layer-V pyramidal cells in V1, area TE, and area 12 of five monkeys at different ages (2D, 3W, 3.5M, 4.5Y; Figure 2, Table 1). A total of 21,939 individual dendritic spines were drawn and tallied. Both basal dendrites and spines of layer-V pyramidal cells had area-specific growth profiles. The profiles were distinct from those previously reported for layer-III pyramidal cells in the same areas (Elston et al., 2009, 2010a).

### Basal dendritic field area

Layer-V pyramidal cells in each examined area exhibited distinct developmental changes in their basal dendritic field areas. At 2D, those in V1 (mean ± standard deviation [SD]; 1.27 ± 0.59 × 10^5^ μm^2^), area TE (0.96 ± 0.33 × 10^5^ μm^2^), and area 12 (1.05 ± 0.32 × 10^5^ μm^2^) were not different from each other (Figure 3A; *p* = 0.12; Kruskal-Wallis test). At 4.5Y, the dendritic trees of pyramidal cells in area 12 were the largest (1.40 ± 0.43 × 10^5^ μm^2^) followed by those in area TE (0.81 ± 0.25 × 10^5^ μm^2^), which were larger than those in V1 (0.40 ± 0.19 × 10^5^ μm^2^) (*p* = 6.9 × 10^−13^; Kruskal-Wallis test; *p* < 10^−4^ for area 12 vs. area TE, and area TE vs. V1; *post-hoc* Mann-Whitney's U-test). This difference resulted from two trends: a decrease in the size of the dendritic trees in V1 with age (*p* = 5.1 × 10^−9^; Kruskal-Wallis test) and an increase in their size in area 12 over the same period (*p* = 7.2 × 10^−5^). The dendritic trees of cells in area TE were similar in size for 2D and 4.5Y (*p* = 0.11; *post-hoc* Mann-Whitney's U-test).

**Figure 4:**
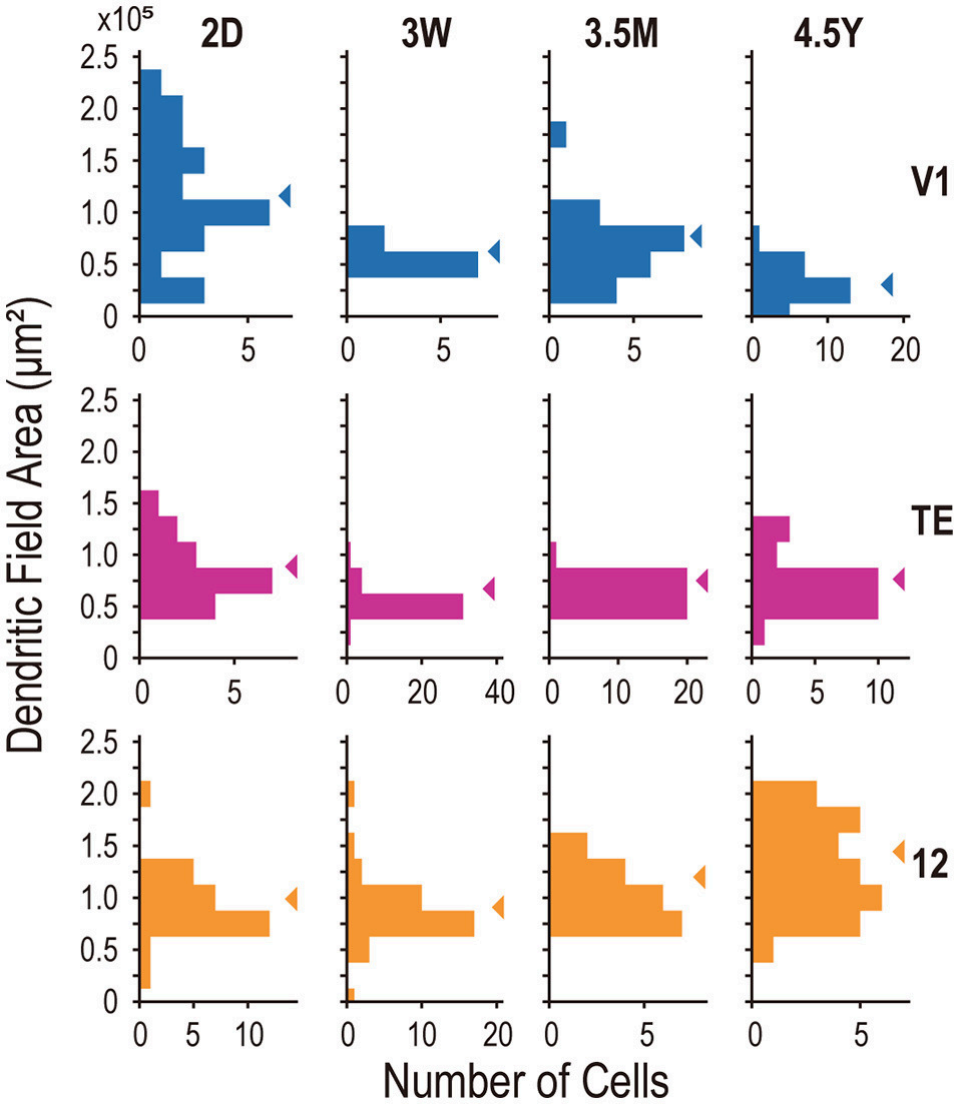
**Frequency histograms of the basal dendritic field area of layer V neurons from areas V1, TE, and 12**. Arrowheads indicate the medians.

The area-specific developmental changes in dendritic tree size appear to result from disappearance of V1 cells with large dendritic fields after 3W and appearance of cells with large dendritic fields in the area 12 of adults (Figure 4). The variance of dendritic field area significantly differed across ages in all three areas (*p* = 3.2 × 10^−5^, 1.5 × 10^−4^, and 0.027 for V1, area TE, and area 12, respectively; Brown-Forsythe test for comparison of variance across the groups). In V1, the distribution of dendritic field area across neurons was broader at 2D (*SD* = 0.59 × 10^5^ μm^2^) than at 3W, 3.5M, or 4.5Y (*SD* = 0.75 × 10^5^, 0.34 × 10^5^, and 0.19 × 10^5^ μm^2^; *p* < 10^−4^, *p* = 0.018, and *p* < 10^−4^, for 2D vs. 3W, 3.5M, and 4.5Y, respectively; *post-hoc F*-test). V1 neurons with dendritic field area larger than 1.0 × 10^5^ μm^2^ were dominant at 2D, but were very few at later ages. The distribution in area TE was broader at 2D (0.33 × 10^5^ μm^2^) than at 3W or 3.5M (*SD* = 0.13 × 10^5^ μm^2^, 0.11 × 10^5^; *p* < 10^−4^), but similar to the distribution at 4.5Y (0.25 × 10^5^ μm^2^; *p* = 0.25). In area 12, neurons with large dendritic fields dominated at 4.5Y compared to the other ages (mean ±*SD* = 1.05 ± 0.32 × 10^5^ μm^2^ at 2D, 0.98 ± 0.32 × 10^5^ μm^2^ at 3W, 1.16 ± 0.25 × 10^5^ μm^2^ at 3.5M, 1.40 ± 0.43 × 10^5^ μm^2^ at 4.5Y; *p* = 0.0013, 0.001, and 0.045 for 2D vs. 4.5Y, 3W vs. 4.5Y, and 3.5M vs. 4.5Y, respectively; *post-hoc* Mann-Whitney's U-test).

### Total length of basal dendrites

Basal dendritic length was constant across ages in V1, and increased in area TE and area 12 during development (*p* = 0.058 in V1; *p* = 1.4 × 10^−4^ in area TE; *p* = 3.4 × 10^−5^ in area 12; Kruskal-Wallis test among ages). At 2D, the length was similar between the three areas (mean ±*SD* = 3856 ± 1769 μm in V1; 3713 ± 1116 μm in area TE; 3735 ± 978 μm in area 12; *p* = 0.99; Kruskal-Wallis test). At 3W the total length of dendrites slightly decreased in V1 (mean ±*SD* = 3112 ± 510 μm in V1), whereas those in area TE and area 12 increased (4743 ± 777 μm in area TE; 4874 ± 1317 μm in area 12; *p* = 0.0015 in area TE [2D vs. 3W]; *p* = 0.0004 in area 12 [2D vs. 3W]; *post-hoc* Mann-Whitney U-test), showing inter-area difference (*p* = 1.6 × 10^−4^; Kruskal-Wallis test). At 4.5Y, the difference in the total dendritic length became further obvious; 2622 ± 1118 μm in V1, 4398 ± 982 μm in area TE, and 5245 ± 962 μm in area 12 (*p* = 8.7 × 10^−10^ in 4.5Y; Kruskal-Wallis test). Net changes between 2D and 4.5Y were −1234 μm for V1 (*p* = 0.03; *post-hoc* Mann-Whitney U-test), +685 μm for area TE (*p* = 0.068), and +1,510 μm for area 12 (*p* = 6.0 × 10^−6^) (Figure 3B).

### Cell-body size

**Figure 5:**
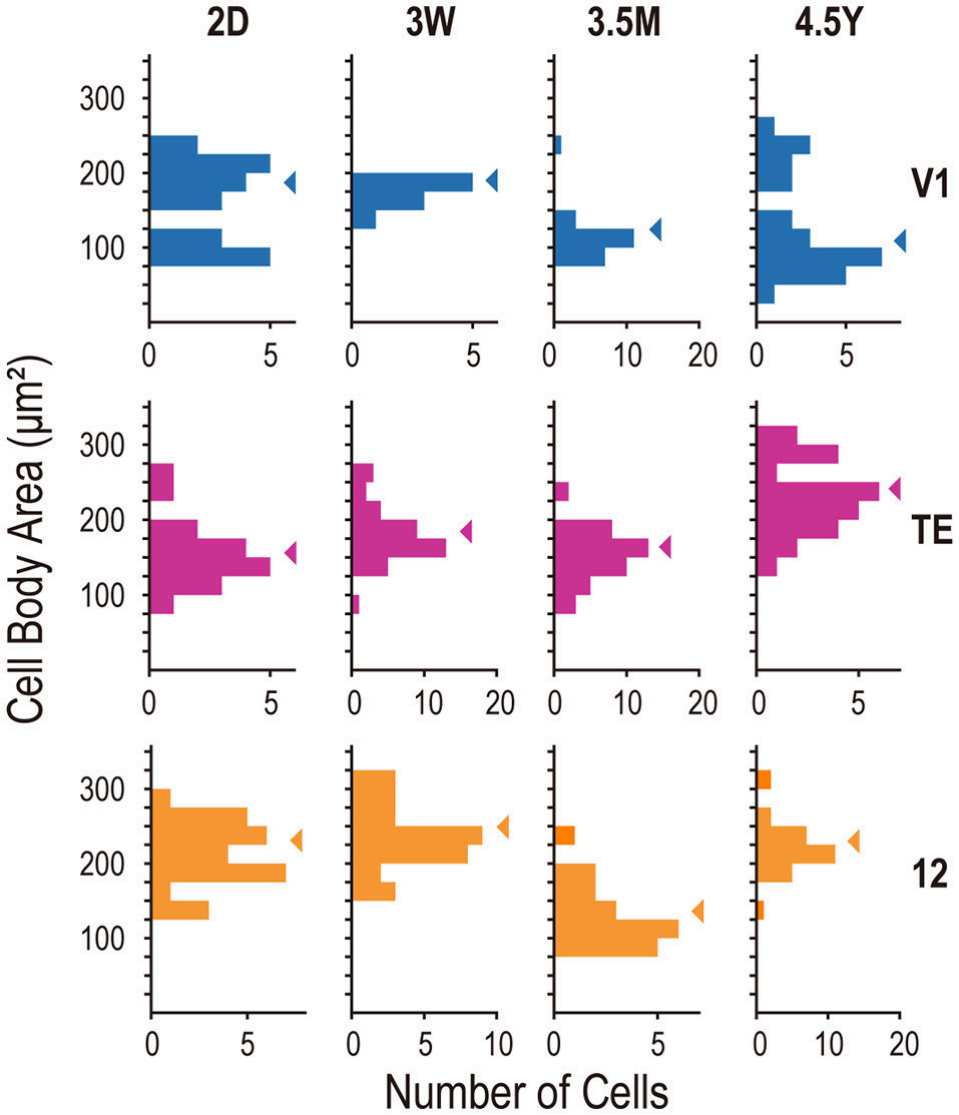
**Frequency histograms of cell-body size of layer-V neurons from areas V1, TE, and area 12**. Arrowheads indicate the medians.

We plotted histograms of cell-body area to examine whether the samples in each area consisted of mixed cell populations. The distributions did not have statistically significant multiple peaks in any area/age combination (*p* > 0.055; Hartigan's dip test). The mean cell-body area differed across ages in all three areas (*p* = 0.0038, 7.0 × 10^−9^, and 2.5 × 10^−9^, for V1, area TE, and area 12, respectively; Kruskal-Wallis test). In V1, the mean values at 2D (mean ± SD; 181 ± 62 μm^2^) and 3W (184 ± 13 μm^2^) were larger than the mean value at 3.5M (127 ± 31 μm^2^) (*p* < 0.01; *post-hoc* Mann-Whitney U-test). In area TE, the mean cell-body area was largest at 4.5Y (246 ± 48 μm^2^) than those at 2D (166 ± 44 μm^2^), 3W (194 ± 37 μm^2^), and 3.5M (167 ± 34 μm^2^) (*p* < 0.01). In area 12, The mean cell-body area was smallest at 3.5M (142 ± 41 μm^2^) than those at 2D (225 ± 42 μm^2^), 3W (262 ± 58 μm^2^), and 4.5Y (239 ± 42 μm^2^) (*p* < 0.01) (Figure 5).

We further examined correlation between the cell-body size and the total dendritic length in each area/age group of cells. If the cell-body size is mainly determined by the metabolic demand of sustaining dendritic arbors, cells with longer total length of dendrites would have larger cell-body. In 4 out of 12 combinations (3 areas × 4 ages), the cell-body size positively correlated to the dendritic length (2D-V1, *r*_*s*_ = 0.744, *p* = 0.0009; 2D-TE, *r*_*s*_ = 0.679, *p* = 0.043; 3W-12, *r*_*s*_ = 0.79, *p* = 4.7 × 10^−6^; 4.5Y-V1, *r*_*s*_ = 0.858; *p* = 2.3 × 10^−5^; *r*_*s*_: Spearman's rank correlation coefficient; *p*-value was Bonferroni corrected). We found no significant correlation in the other combinations. When examined for the data collapsed across all cells, there was a relatively strong correlation; the bigger the cell body, the longer the basal dendrites (*r*_*s*_ = 0.497; *p* = 8.7 × 10^−21^).

### Branching patterns of basal dendrites assessed by sholl analysis

The number of peak intersections yielded by the Sholl profile of layer-V pyramidal cell dendritic trees at 2D was greatest in area TE (mean ± SD; 25.1 ± 6.6), followed by area 12 (23.3 ± 5.9), and V1 (19.6 ± 7.2) (Figure 3C). Peak branching complexity was observed in the dendritic trees of pyramidal cells in V1 and area TE at 3W, with area TE complexity being considerably higher than that in V1 (38.4 ± 6.0 and 26.0 ± 5.5, respectively). In area 12, branching complexity in these cells was highest at 4.5Y (31.8 ± 6.2). In the adult, pyramidal cells in TE (29.8 ± 8.0) and area 12 (31.8 ± 6.2) had similar numbers of branch points, which were greater than the numbers of branches in V1 (24.5 ± 6.9). Statistical analysis revealed that the peak number of intersections in the Sholl profiles significantly differed across age groups in each area (*p* = 1.7 × 10^−3^, *p* < 1 × 10^−5^, and *p* < 1 × 10^−5^, for V1, area TE, and area 12, respectively; Kruskal-Wallis test).

### Fractal dimension

Fractal dimension—an indicator of dendritic complexity (Elston and Jelinek, 2001)—increased in all three areas (*p* = 0.031, 1.2 × 10^−7^, and 2.3 × 10^−5^; for V1, TE and 12, respectively; Kruskal-Wallis test). At 2D, the fractal dimension was similar between V1 (1.109 ± 0.037), area TE (1.134 ± 0.042), and area 12 (1.121 ± 0.033) (*p* = 0.27). The fractal dimension in area TE (1.184 ± 0.021; *p* = 0.0001; *post-hoc* Mann-Whitney's U-test [2D vs. 3W]) and area 12 (1.158 ± 0.030; *p* = 0.0001; *post-hoc* Mann-Whitney's U-test [2D vs. 3W]) increased at 3W and difference between the areas emerged (*p* = 2.2 × 10^−5^; Kruskal-Wallis test). The fractal dimension of area TE then decreased at 3.5M (1.154 ± 0.019; *p* = 6.9 × 10^−8^; *post-hoc* Mann-Whitney's U-test). At 4.5Y, the fractal dimension of area TE (1.165 ± 0.025) and area 12 (1.163 ± 0.026) was larger than that of V1 (1.135 ± 0.038) (*p* = 4.2 × 10^−4^; Kruskal-Wallis test; *p* = 0.0006, 0.0008, 0.83 for V1 vs. TE, V1 vs. 12, and TE vs. 12, respectively; *post-hoc* Mann-Whitney's U-test). Thus, the complexity of dendritic branching patterns were similar at birth between the three areas, and became different in adults with area TE and area 12 being more complex than V1 (Figure 3D).

### Spine densities of the basal dendrites

**Figure 6:**
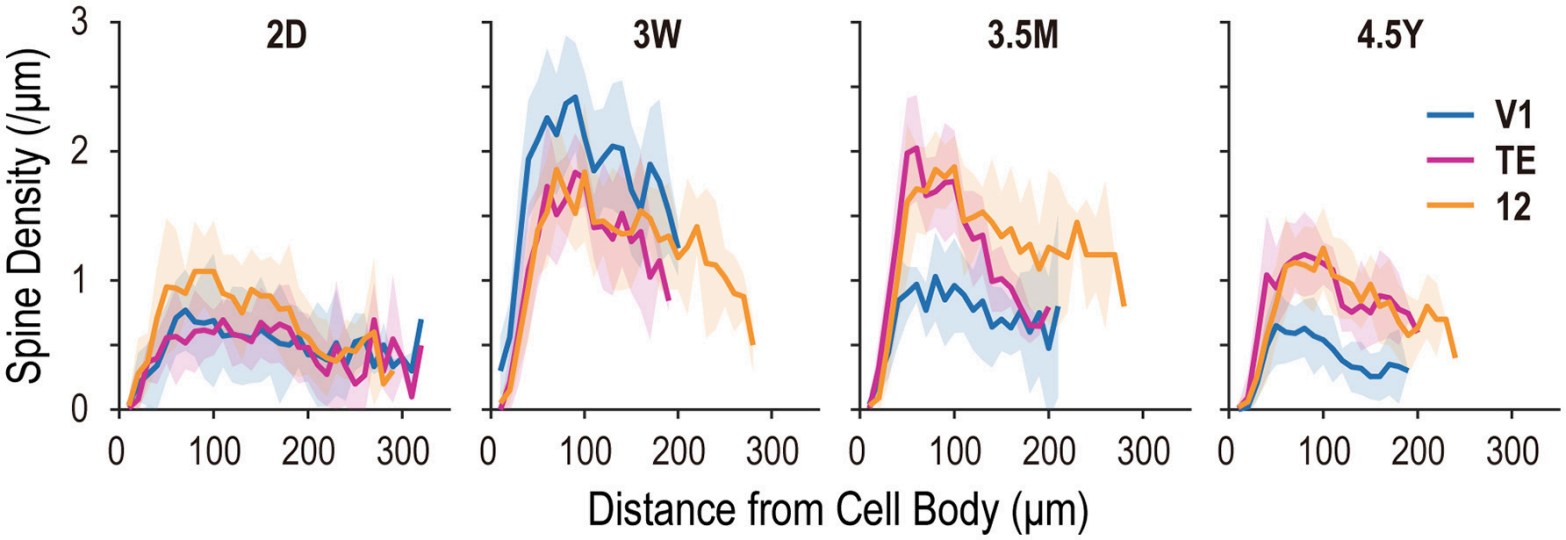
**Spine density plots for the basal dendrites of layer-V pyramidal cells, sampled from areas V1, TE, and area 12 at 2 days (2D), 3.5 months (3.5M), and 4.5 years (4.5Y)**. Each profile indicates dendritic spine density along the dendritic extent. Shaded areas indicate standard deviation.

At 2D, peak spine density (spines/μm) along dendritic segments in area 12 (mean ± SD; 1.07 ± 0.39) was greater than that observed in V1 or area TE (0.77 ± 0.31 and 0.70 ± 0.16, respectively) (Figure 6; see also Figure 2B). The greatest peak spine density in V1 was observed at 3W (2.42 ± 0.42), which was lower at each successive time point (3.5M: 1.03 ± 0.34; 4.5Y: 0.65 ± 0.30). The greatest peak spine densities in TE and area 12 were observed at 3W (1.85 ± 0.30 and 1.86 ± 0.41, respectively) and 3.5M (2.04 ± 0.41 and 1.88 ± 0.24, respectively), being approximately 30% higher than those observed at 4.5Y (1.21 ± 0.33 and 1.25 ± 0.31, respectively) (Figure 6). At 4.5Y, the peak densities in TE and area 12 were about 1.9 times higher than that in V1 (0.65 ± 0.30). A statistical test revealed these differences in spine density were significant across ages (*p* < 10^−5^; for V1, TE, and area 12; Kruskal-Wallis test). Thus, all three areas attained peaks in spine density as early as 3W. At 3.5M, density had already substantially decreased in V1, whereas it was still at peak levels in TE and area 12.

**Figure 7:**
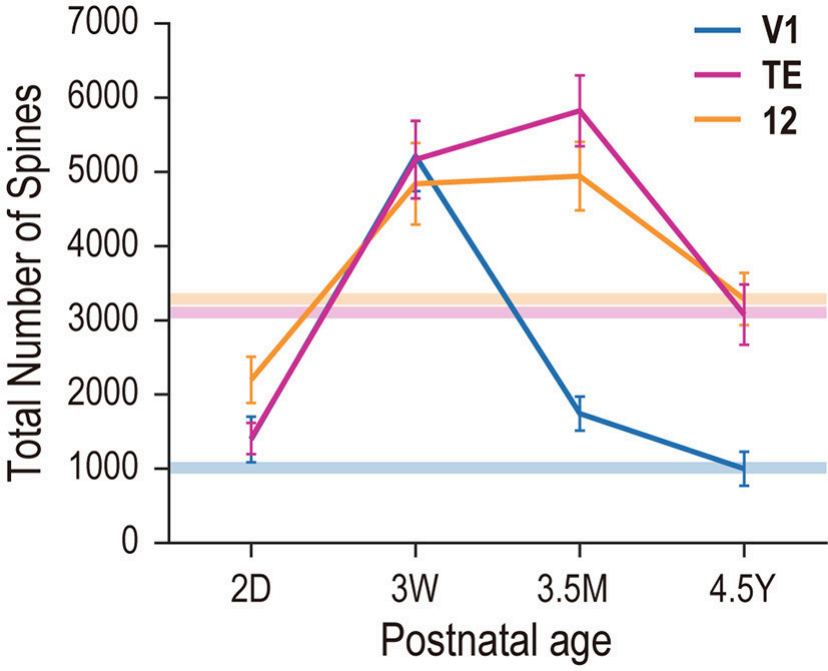
**Total number of dendritic spines in the basal dendritic tree of the “average” cell in each cortical area and age**. Horizontal bars with different colors indicate the adult levels for areas V1, TE, and area 12.

Estimates of the total number of spines in the basal dendritic tree of the “average” pyramidal cell revealed striking differences in postnatal changes between the three areas. At 2D, cells in area 12 were considerably more spinous (2198) than those in V1 (1395) or area TE (1408) (Figure 7). By 3W, cells in V1, area TE and area 12 had 2–3 times more spines, with levels evening out across areas (4840, 5167, 5214, respectively). At 3.5M, the numbers of spines in area 12 (4943) and area TE (5825) had increased, while that in V1 was reduced by about 60% (1742). By 4.5Y, the numbers of spines in the dendritic tree of the “average cell” in area 12 (3287) and TE (3076) were approximately 30% less, while in V1 the number had decreased by 40% (1000) (Figure 7). Thus, the total number of spines in the basal dendrites of V1 pyramidal cells quickly decreased after the peak at 3W, and continued to decrease into the 5th year of life, resulting in adults having fewer spines than newborn monkeys. In contrast, area TE and area 12 exhibited broader peaks that spanned from 3W to 3.5M, with adults having more spines than newborns.

## Discussion

**Figure 8:**
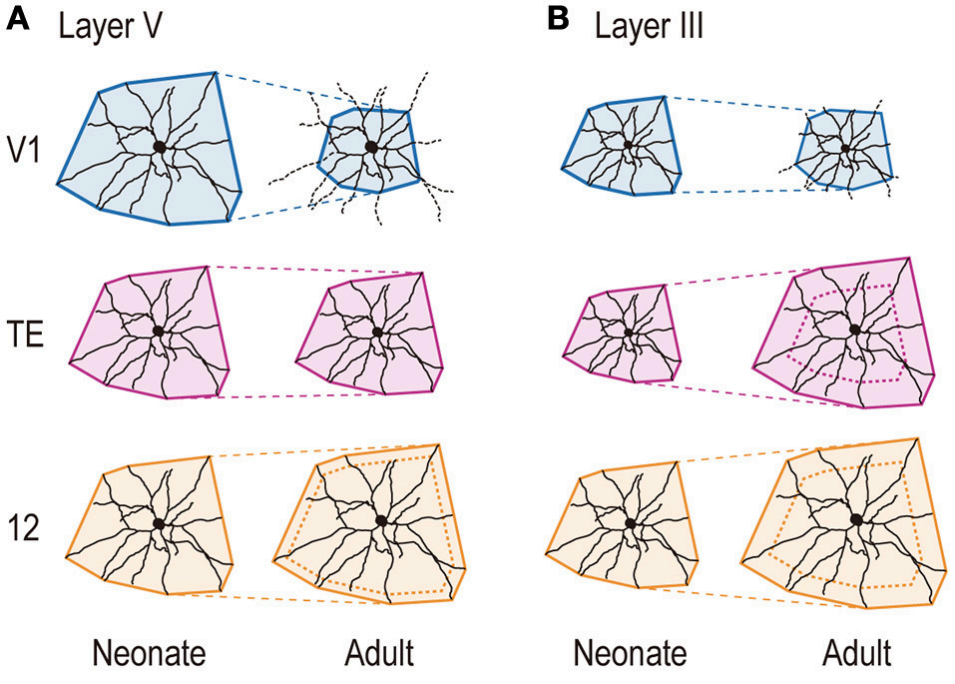
**Schematic illustration showing area-specific and layer-specific developmental changes of pyramidal cell dendrites**. The cells are drawn proportional in size to the mean dendritic field areas of corresponding area/age groups. Layer-V pyramidal cells in V1 retract basal dendritic arbors postnatally, whereas those in area TE remain constant in size, and those in area 12 become larger, increasing the size of their dendritic field areas **(A)**. Layer-III basal dendritic field areas of pyramidal cells in area TE and area 12 increase with age, whereas those of V1 cells decrease **(B)**. The scheme for layer V is based on the current results, and that for layer III is based on Elston et al. (2009, 2010a).

In the present investigation, we studied the basal dendritic tree morphology of layer-V pyramidal cells in V1, cytoarchitectonic area TE, and Walker's area 12 in macaque monkeys that ranged in age from 2 days to 4.5 years. The main aim of the study was to find differences, if they exist, in the developmental profiles of layer-V pyramidal cells across these cortical areas. The profiles were indeed area-specific. Layer-V pyramidal cells in V1 retracted basal dendritic arbors postnatally, whereas those in area TE remained constant in size, and those in area 12 became larger, increasing the size of their dendritic field areas (Figures 3, 4, 8A). Layer-V pyramidal cells in V1 exhibited peak spine density and the greatest number of spines at 3W, whereas area TE and area 12 maintained the greatest density and numbers of spines over a longer period, spanning 3W and 3.5M (Figures 6, 7). This early maturation distinguishes layer V from layer III in which pyramidal cells in all three areas concurrently reach peak density and total number of spines at 3.5M (Elston et al., 2009, 2010a).

### Area-specific postnatal changes in pyramidal cell morphology

The results revealed relative differences in the development of pyramidal cell dendritic trees among the three cortical areas. Specifically, basal dendritic trees of cells in V1 were largest at 2D and continued decreasing in size into adulthood, those in TE were similar in size at 2D and 4.5Y, and those in area 12 increased in size from birth to adulthood (Figure 3). Peak spine density was greatest at 3W in V1, and lasted from 3W to 3.5M in area TE and area 12 (Figure 6). Estimates of the total number of spines in the dendritic trees revealed that pyramidal cells in V1 attained their greatest number of spines at 3W, and these were subsequently pruned by 3.5M and moreso by 4.5Y (Figure 7). Cells in adult V1 contained less than 20% the number of spines observed at the 3-week-old peak. Cells in area TE and area 12 attained their greatest numbers of spines at 3.5M, and those in adults contained approximately 40% (TE) and 50% (area 12) of their peak numbers. Of note, cells in V1 lose more spines than they grow following the onset of visual experience, whereas those in area TE and area 12 grow more spines than they lose during the same period (Figure 7; compare the colored horizontal bars that denote adult levels for the three areas at 2D). These data provide further evidence for regional variation in pyramidal cell development across different cortical areas, including infragranular cells.

While in general peaks in synaptogenesis may occur approximately 3.5 months after birth across cortical areas and layers (Rakic et al., 1986; Bourgeois and Rakic, 1993; Bourgeois et al., 1994), the basal dendritic tree spine count of layer-V cells in V1 was greatest at 3W, followed by a net decrease of approximately 60% by 3.5M (Figure 7). In area 12, dendritic trees continued to grow from 2D into adulthood. These larger and more branched dendritic trees are replete with spines, which presumably accommodate functioning asymmetrical synapses (e.g., Arellano et al., 2007), suggesting that additional functional synapses are grown beyond 3.5M into adulthood.

In the present analysis, we did not consider the heterogeneity of pyramidal cells in layer V. Layer V is composed of two sublayers, VA and VB (Brodmann, 1909). Cell density is greater in VA than VB (Figures 1B–D), and cell bodies are larger on average in layer VB than in layer VA. We did not distinguish these sublayers. Our samples from each area and age might thus include pyramidal cells with different ratios of VA and VB neurons. In addition to the sublayers, layer V contains subtypes of pyramidal cells that differ in their projection targets (i.e., cortical vs. subcortical, O'Leary and Koester, 1993). Subcortically projecting neurons may have larger cell bodies and thicker and more extensive apical dendrites than cortically projecting neurons (see Feldman, 1984 for review; Kim et al., 2015). Although the differences were not statistically significant, an inspection of cell body-size distribution in V1 suggests that two subpopulations, large cells and smaller cells, might exist in our V1 dataset (Figure 5). The data at 2D and 4.5Y may include both subpopulations, whereas the data at 3W and 3.5M might include only one of them. Despite these caveats, we believe that the decrease in dendritic field area in V1 was genuine because the data at 2D and 4.5Y include both subpopulations and is consistent with previous observations in layer III.

In addition to dendrites, pyramidal cell axons also exhibit area-specific morphology and developmental changes. The size, spacing, and maximal extent of the terminal patches belonging to intrinsic horizontal axons of layer-III pyramidal cells are greater in area TE than in V1 (Fujita and Fujita, 1996; Tanigawa et al., 2005). These characteristics are already evident 1 week after birth, and are refined by postnatal development into the adult-type phenotype (Wang et al., 2016). The number and distribution of patches, but not the inter-patch distance, gradually decrease with age in V1, suggesting that the furthest patches in infants are later pruned. In contrast, this change in the number and extent of patches does not occur in area TE. Future studies are required to determine how genetic and epigenetic mechanisms interact to produce regional specialization of dendrites and axons in the primate brain (Whitford et al., 2002; Malyshevskaya et al., 2013; Sasaki et al., 2014a,b; Bakken et al., 2016).

### Layer-specific development of pyramidal cells: comparison between layer V and layer III

Previously, we showed that basal dendritic field area of layer-III pyramidal cells in area TE and area 12 increased with age, whereas those of V1 cells decreased (Figure 8B). In contrast, while the trends in layer V were similar to those in layer III for V1 and area 12, dendritic field area remained relatively unchanged over time in area TE (Figure 8A).

Overproduction and pruning of spines was a common feature between the two layers, but the time course was different. In layer III, spine density peaked at 3.5M in all three areas (Elston et al., 2009), whereas it peaked earlier in layer V (3W in V1 and from 3W to 3.5M in area TE and area 12; Figure 6). Inter-area differences in spine density became obvious only after 7 months in layer III. In contrast, spine density in layer-V cells exhibited inter-area differences throughout development. Thus, within each area, postnatal pyramidal cell development differs between layer III and layer V. However, the overall changes in the total number of spines were similar between layer III and layer V in that pyramidal cells in both layers showed increasing numbers of dendritic spines with age in area TE and area 12 and decreasing numbers in V1 (Elston et al., 2009 for layer III; Figure 7 for layer V). The absolute number of spines, however, differ between layers V and III.

### Inter-individual differences in pyramidal cell morphology

In general, the variation in age/gender/area/layer/topography matched pyramidal cell structure among individuals is markedly less than differences observed between cortical areas within an individual or within a given cortical area at different developmental ages. Data sampled from a specific cortical area/layer in a number of different individuals have been reported for each individual in the galago (Elston et al., 2005), the marmoset (cf. Elston et al., 1996, 1999b), the South American Agouti (Elston et al., 2006b) and macaque (Oga et al., 2016), revealing highly conserved structure in age/gender/area/layer/topography matched pyramidal cells among individuals. The exception, thus far, is the granular prefrontal cortex where studies in the baboon, vervet monkey and macaque monkey reveal inter-individual variation in pyramidal cell structure among age/gender/area/layer/topography matched pyramidal cell structure not observed in any other cortical region (Elston et al., 2011a—in particular, see Figure 7).

In the current dataset, our samples at 4.5Y were obtained from two monkeys. We evaluated an inter-individual difference by comparing the total dendritic length, a factor that strongly influences the total number of spines. There was a difference between the two monkeys in V1, whereas no difference was found in area TE and area 12. The ratio of the total dendritic length between the two monkeys were 1.98 in V1 (1,902 vs. 3,775 μm; *p* = 5.5 × 10^−5^; Mann-Whitney's U-test), 1.14 in area TE (4,144 vs. 4,743 μm; *p* = 0.16), and 1.08 in area 12 (5,368 vs. 4,972 μm; *p* = 0.21). The inter-individual difference in V1 may be caused by a difference in cortical depth sampled (e.g., sub-layer VA or VB), as suggested by the bimodal distribution of cell-body size (see Figure 5). When compared between the two monkeys, the cell body size was different in V1 (MF1, 92.6 ± 17.9 μm^2^, *n* = 16; CI15, 216.7 ± 45.5 μm^2^, *n* = 10; *p* = 2.8 × 10^−5^), but similar in area TE (MF1, 260.4 ± 43.9 μm^2^, *n* = 15; CI15, 225.3 ± 47.5 μm^2^, *n* = 11; *p* = 0.049) and area 12 (MF1, 241.4 ± 45.2 μm^2^, *n* = 20; CI15, 234.5 ± 35.3 μm^2^, *n* = 9; *p* = 0.80). In contrast to the individual differences of the total dendritic length, the largest change over the postnatal development was 1.47 in V1 (3,856 [2D] vs. 2,622 [4.5Y]), 1.34 in area TE (4,743 [3W] vs. 3,713 [2D]), 1.45 in area 12 (3,735 [2D] vs. 5,245 [4.5Y]), showing that the developmental change in area TE and area 12 was larger than the individual difference.

### Comparative development of pyramidal cells in human and other primate species

A continuous postnatal decrease in the size of the basal dendritic field in layer-III and layer-V pyramidal cells during development is a striking feature of the primary sensory cortices in cynomolgus monkeys (layer III in V1, Elston et al., 2010a; layer V in V1, current study; layer III in primary auditory cortex, Elston et al., 2010b). In V1 of humans and other non-human species, there is a period of postnatal growth before this decrease. The age at which this decrease begins in these species varies from 3W to 15W in layer IIIB in southern pig-tailed macaques (Boothe et al., 1979), and 12–24 months in human layer V (Becker et al., 1984). In a New World primate (marmoset), layer III pyramidal cells in V1 do not exhibit postnatal decrease in the size of their dendritic arbors (Oga et al., 2013).

Pyramidal cells in prefrontal cortex (both ventrolateral and dorsolateral) grow their dendrites from infancy to adulthood. This continuous growth is a common feature in marmosets (layer III in area 12; Oga et al., 2013), cynomolgus monkeys (layer III and V in area 12; Elston and Fujita, 2014 and the current study) and humans (layer III in BA9 and BA46: Koenderink et al., 1994; layer V in BA9 and BA46: Koenderink and Uylings, 1995; layers III and V in BA9: Petanjek et al., 2008).

At birth, dendritic branches of pyramidal cells in human cerebral cortex are on average longer in layer V than in layer III (Becker et al., 1984 for V1; Mrzljak et al., 1992 for BA9 and BA46; Petanjek et al., 2008 for BA9). Pyramidal cells in layer V are born earlier, and might start to grow dendrites earlier than cells in layer III (Mrzljak et al., 1992). Dendritic field area in cynomolgus monkeys at 2D was also larger in layer V than in layer III in each area (Figures 8A,B; comparison between current study and Elston et al., 2009, 2010a).

Petanjek et al. (2008) reported that layer III pyramidal cells in human BA9 undergo development in two stages. As discussed above, dendrites are less developed in length at birth in layer III than in layer V. Within 1 month, dendrites in layer III rapidly grow and catch up to the size of dendrites in layer V. After this initial growth, layer III neurons maintain their size during the 3–16 months after birth (steady period). At this point (around age 2.5 years), the second growth stage begins. In the macaque monkey, area 12 pyramidal cells in both layers III and V maintain the same dendritic field size between 2D and 3W, but then show rapid dendritic growth from 3W to 3.5M (Elston et al., 2009; current study). The period from 2D to 3W in monkeys may correspond to the steady period observed in human BA9. The rapid growth of layer-III dendrites is accompanied by peak synaptogenesis and spinogenesis in both human (2–2.5 years; Huttenlocher and Dabholkar, 1997 for middle frontal gyrus; Petanjek et al., 2011 for BA9) and macaque (2–4 months; Rakic et al., 1986; Bourgeois and Rakic, 1993; Bourgeois et al., 1994; Elston et al., 2009).

### Changes of pyramidal cell dendrites and spines in older age

Dendritic length and the number of spines gradually decrease in older age. Layer III pyramidal cells in human BA10 and BA18 have shorter basal dendrites and a smaller number of dendritic spines in an older group (> 50-year old) than in a younger group (≤ 50-year old) (Jacobs et al., 1997). Spines on basal and oblique dendrites of both layer III and V pyramidal cells in human BA9 also continuously decrease after puberty (Petanjek et al., 2011). In macaque monkeys, the total number of spines in basal dendrites decline from 3,076 at 4.5Y (this study) to 2,112 at 16Y (Elston and Rosa, 2000) in layer V pyramidal cells of area TE, with the magnitude of change roughly corresponding to one S.D.

## Conclusion

The data sampled from layer V provide further evidence that pyramidal cells in different cortical areas are characterized by different growth profiles, and expands the findings in previous reports with regard to regional specialization in layer-III cells. Further, when compared directly with pyramidal cells sampled from layer III of the same animals, the current data reveal that within a given cortical area, pyramidal cells in infragranular layers may have different growth profiles compared with those immediately above in supragranular layers. These different growth profiles result in fundamentally different dendritic trees among different cortical areas and impact dendritic function and neuronal circuits throughout the cortical depth. Further investigation into regional and laminar specializations in developing and mature pyramidal cells, and the functional implications of the structural specialization in human and monkey, will likely yield fruitful insights into human and non-human behavior (see Elston, 2007; Spruston, 2008; DeFelipe, 2011; Elston and Fujita, 2014 for reviews).

## Author contributions

TO, GE, and IF conceived, designed, and performed the experiments. TO analyzed the data. TO, GE, and IF wrote the paper.

## Funding

This work was supported by grants from the Japan Science and Technology Agency (Core Research for Evolutional Science and Technology), the Ministry of Education, Culture, Sports, Science, and Technology (MEXT; JP15H01437, JP16H01673), Osaka University Global COE program, Center for Information and Neural Networks, the National Health and Medical Research Council (210250), the JS McDonnell Foundation. TO was supported by the Japan Society for the Promotion of Science Research Fellowship (JP15J05524).

### Conflict of interest statement

The authors declare that the research was conducted in the absence of any commercial or financial relationships that could be construed as a potential conflict of interest.
